# Supplementary material for: One-carbon metabolic enzymes are regulated during cell division and make distinct contributions to the metabolome and cell cycle progression in Saccharomyces cerevisiae
Source: G3 (Bethesda). 2023 Jan 11;13(3):jkad005. doi: 10.1093/g3journal/jkad005 (PMC9997564; doi:10.1093/g3journal/jkad005)
Supplement: jkad005_Supplementary_Data [file jkad005_supplementary_data.zip › FIGURE S2.pdf]

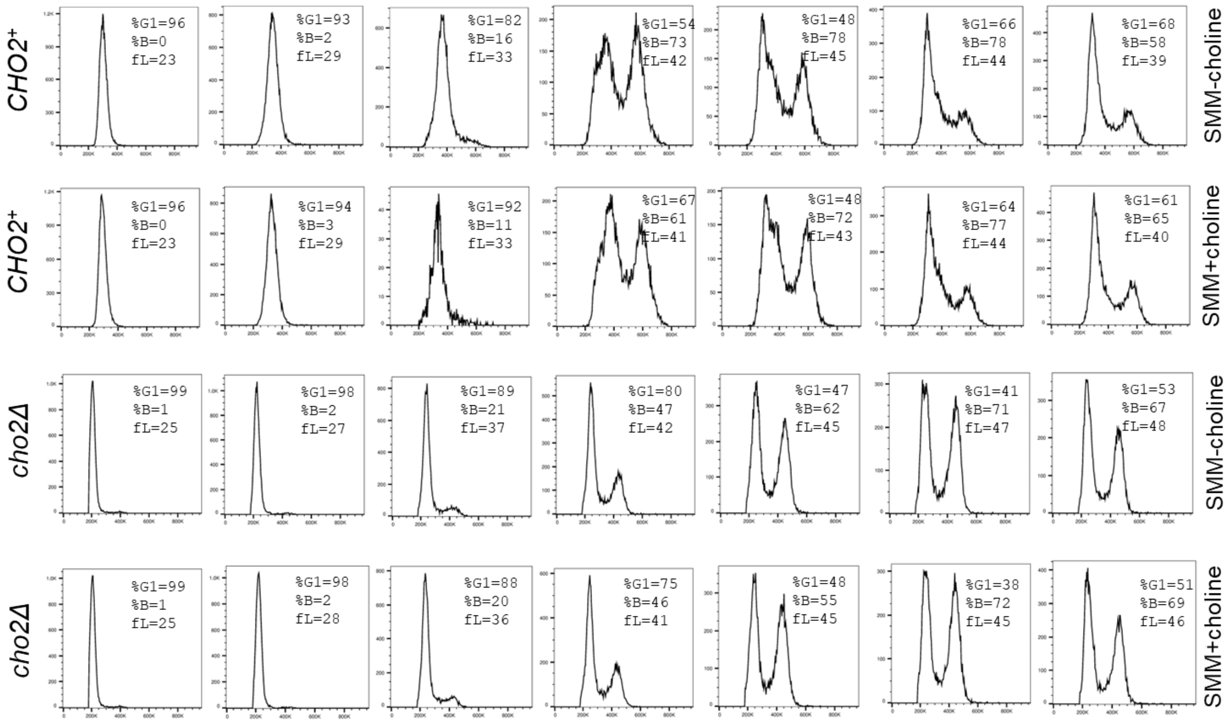

FIGURE S2. DNA content profiles of *cho2Δ* cells during cell division. Synchronous early G1 cultures of the indicated background and medium were obtained by elutriation and allowed to progress in the cell cycle in the medium shown in each case. At regular intervals aliquots were taken and processed for DNA content analysis and flow cytometry. The percentage of budded cells (%B) and cell size (in fL) were recorded (see Materials and Methods). The x-axis on the DNA content histograms represents fluorescence per cell, while on the y-axis are the number of cells.
