# Supplementary material for: One-carbon metabolic enzymes are regulated during cell division and make distinct contributions to the metabolome and cell cycle progression in Saccharomyces cerevisiae
Source: G3 (Bethesda). 2023 Jan 11;13(3):jkad005. doi: 10.1093/g3journal/jkad005 (PMC9997564; doi:10.1093/g3journal/jkad005)
Supplement: jkad005_Supplementary_Data [file jkad005_supplementary_data.zip › FIGURE S3.pdf]

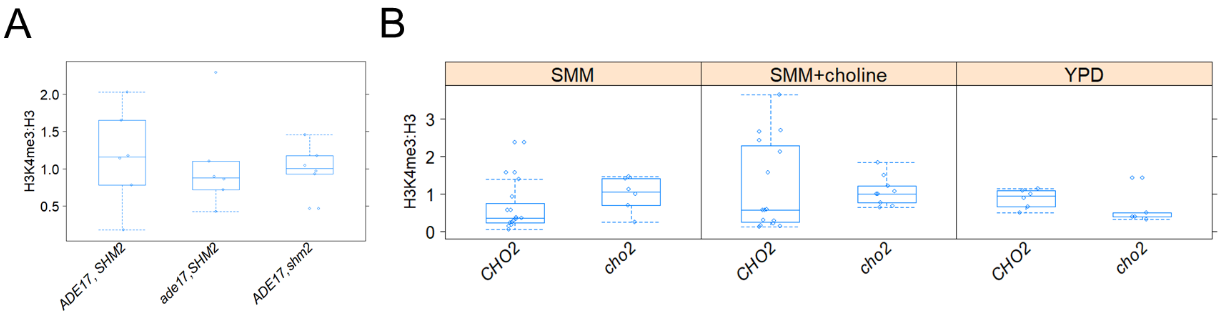

FIGURE S3. Histone methylation levels in 1C mutants. A, Box plots showing the ratio of methylated to total histone H3 (y-axis) of the indicated strains in rich, YPD medium. B, Box plots showing the ratio of methylated to total histone H3 (y-axis) of the indicated strains and media. The values used to generate the graphs are in File S1/Sheet5, based on the immunoblots shown in Figure S4.
