# Supplementary material for: One-carbon metabolic enzymes are regulated during cell division and make distinct contributions to the metabolome and cell cycle progression in Saccharomyces cerevisiae
Source: G3 (Bethesda). 2023 Jan 11;13(3):jkad005. doi: 10.1093/g3journal/jkad005 (PMC9997564; doi:10.1093/g3journal/jkad005)
Supplement: jkad005_Supplementary_Data [file jkad005_supplementary_data.zip › FIGURE S5.pdf]

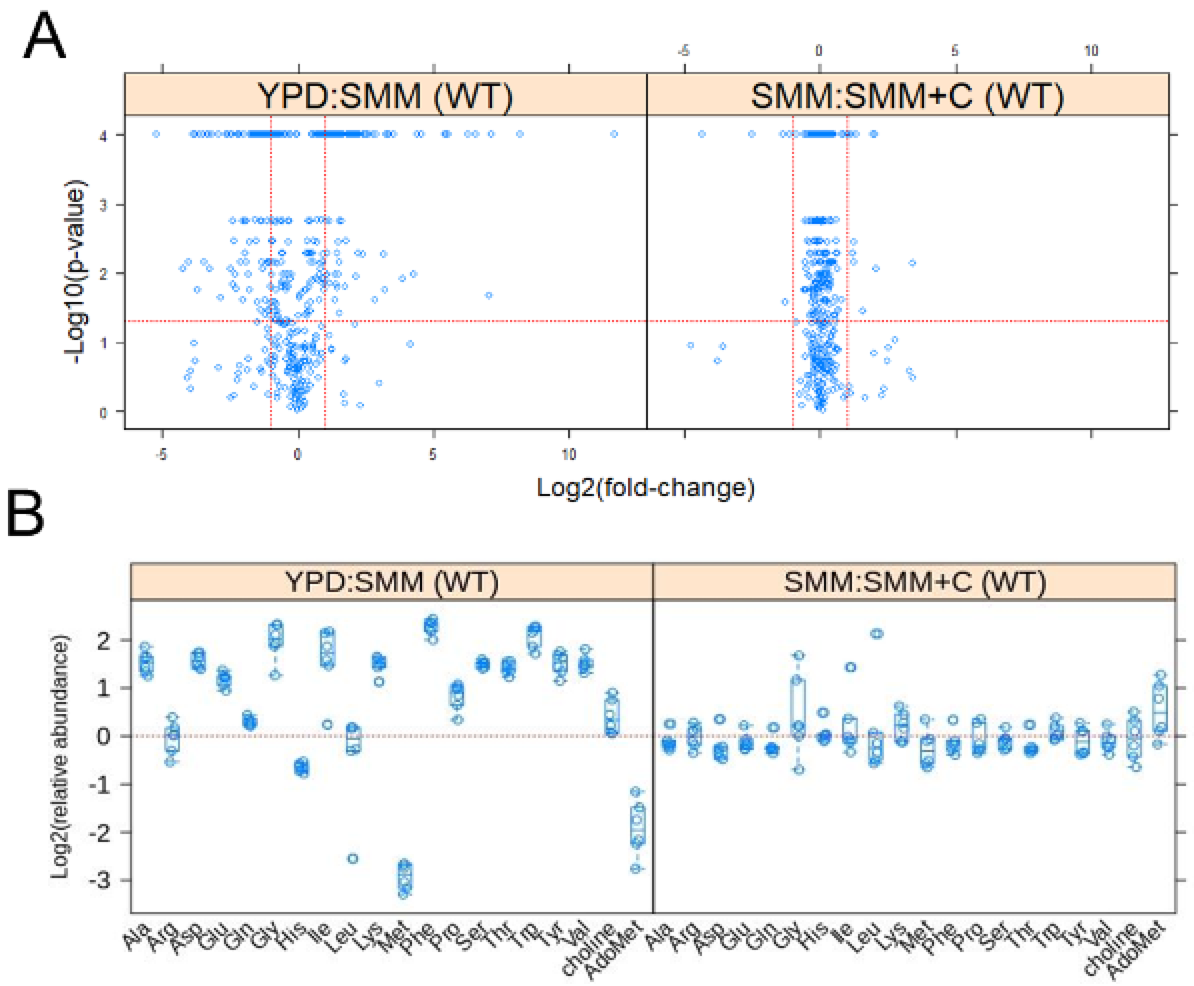

Figure S5. Comparison of metabolite levels in haploid wild type cells cultured in different media. A, Metabolites whose levels changed in the indicated pairwise comparisons were identified from the magnitude of the difference (x-axis; Log2-fold change) and statistical significance (y-axis), indicated by the red lines. The analytical and statistical approaches are described in Materials and Methods. The values used to generate the graphs are in File S1/Sheet6. B, Boxplots of the levels of the amino acids (x-axis) detected in the biogenic amine dataset (see Materials and Methods), shown as Log2-transformed relative abundance between SMM vs. SMM+choline media (y-axis), from six independent samples in each case. The levels of choline and AdoMet (S-adenosylmethionine) from the same measurements are also shown. The values used to generate the graphs are in File S1/Sheet7.
