# Supplementary material for: One-carbon metabolic enzymes are regulated during cell division and make distinct contributions to the metabolome and cell cycle progression in Saccharomyces cerevisiae
Source: G3 (Bethesda). 2023 Jan 11;13(3):jkad005. doi: 10.1093/g3journal/jkad005 (PMC9997564; doi:10.1093/g3journal/jkad005)
Supplement: jkad005_Supplementary_Data [file jkad005_supplementary_data.zip › FIGURE S6.pdf]

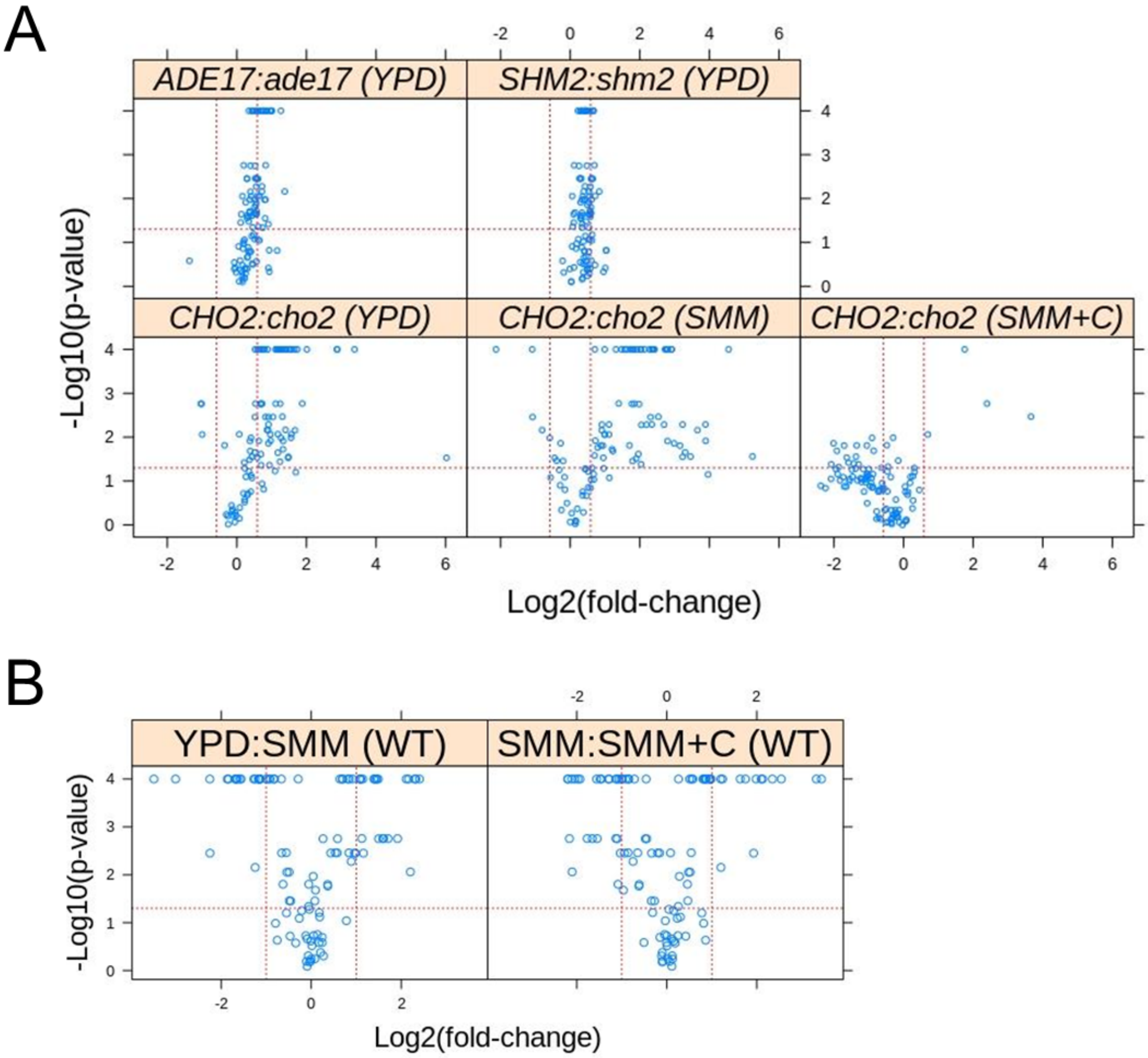

Figure S6. Comparison of complex lipid levels in different strains and media. Complex lipids whose levels changed in the indicated pairwise comparisons were identified from the magnitude of the difference (x-axis; Log2-fold change) and statistical significance (y-axis), indicated by the red lines. The analytical and statistical approaches are described in Materials and Methods. The values used to generate the graphs are in File S1/Sheet8.
