# Supplementary figures and images for: One-carbon metabolic enzymes are regulated during cell division and make distinct contributions to the metabolome and cell cycle progression in Saccharomyces cerevisiae
Source: G3 (Bethesda). 2023 Jan 11;13(3):jkad005. doi: 10.1093/g3journal/jkad005 (PMC9997564; doi:10.1093/g3journal/jkad005)

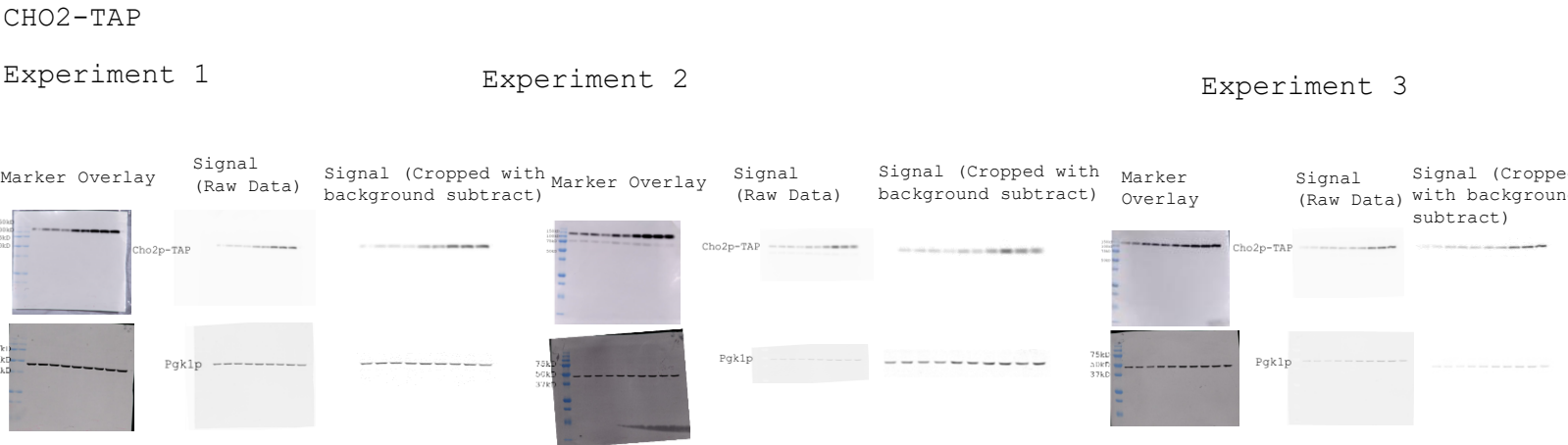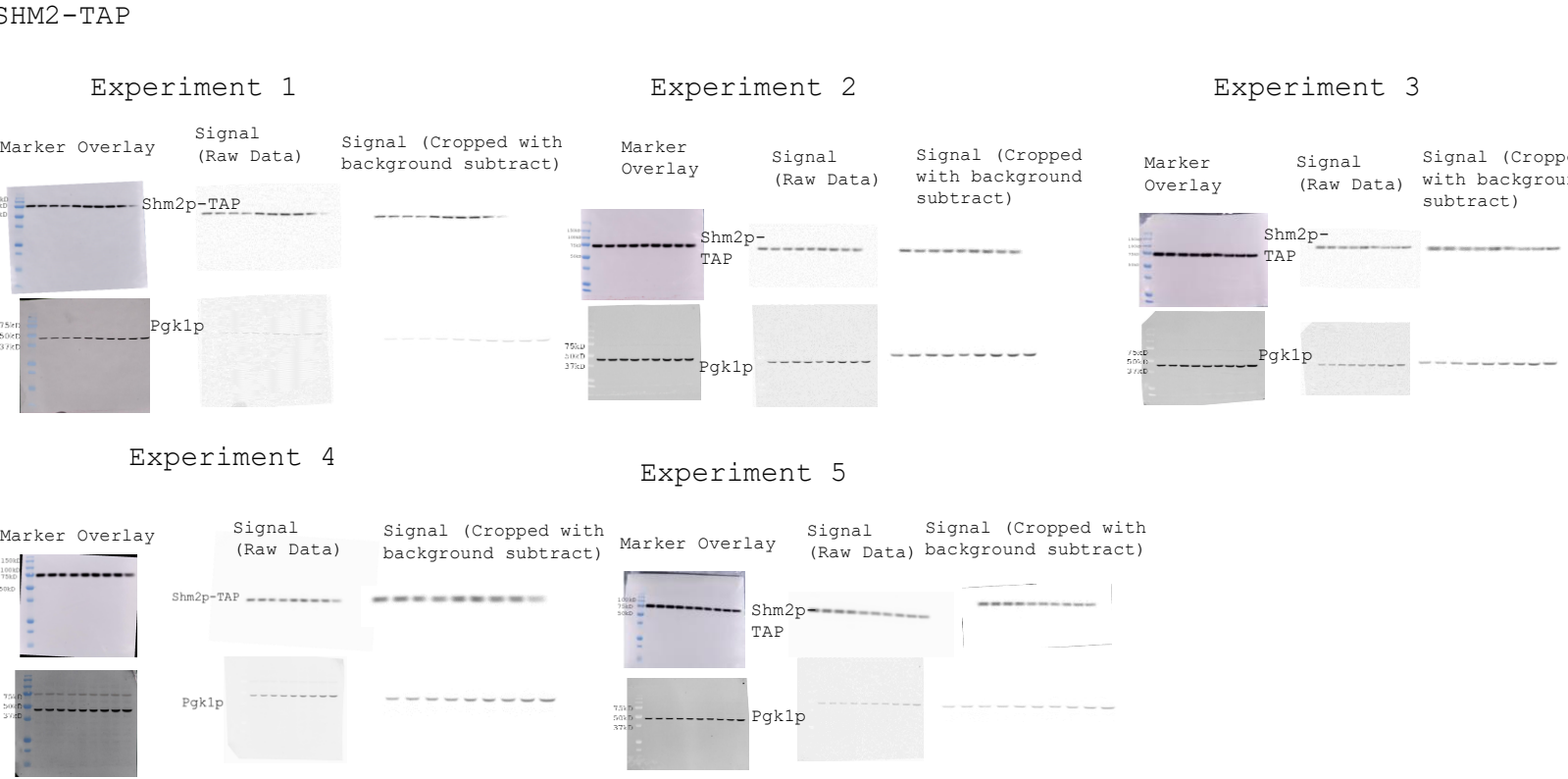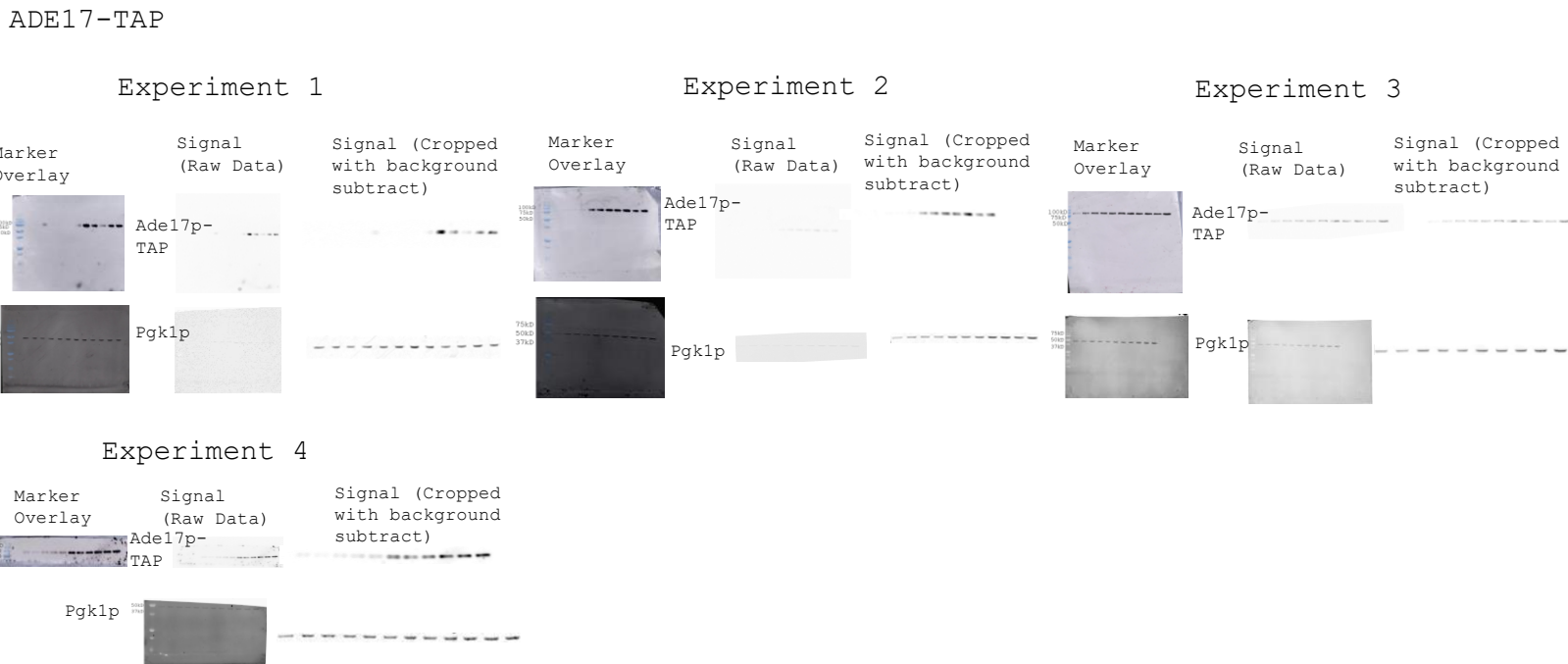

FIGURE S1. Source immunoblots for the protein abundances of Figure 2.

Supplement: jkad005_Supplementary_Data [file jkad005_supplementary_data.zip › FIGURE S1.pdf]
